# Structure-Activity Relationship and Substrate-Dependent Phenomena in Effects of Ginsenosides on Activities of Drug-Metabolizing P450 Enzymes

**DOI:** 10.1371/journal.pone.0002697

**Published:** 2008-07-16

**Authors:** Miao Hao, Yuqing Zhao, Peizhan Chen, He Huang, Hong Liu, Hualiang Jiang, Ruiwen Zhang, Hui Wang

**Affiliations:** 1 Key Laboratory of Nutrition and Metabolism, Institute for Nutritional Sciences, Shanghai Institutes for Biological Sciences, Chinese Academy of Sciences and Graduate School of the Chinese Academy of Sciences, Shanghai, People's Republic of China; 2 Shenyang Pharmaceutical University, Shenyang, People's Republic of China; 3 Drug Discovery and Design Center, Shanghai Institute of Materia Medica, Chinese Academy of Sciences, Shanghai, People's Republic of China; 4 Department of Pharmacology and Toxicology, Division of Clinical Pharmacology, Comprehensive Cancer Center, University of Alabama at Birmingham, Birmingham, Alabama, United States of America; Swiss Tropical Institute, Switzerland

## Abstract

Ginseng, a traditional herbal medicine, may interact with several co-administered drugs in clinical settings, and ginsenosides, the major active components of ginseng, may be responsible for these ginseng-drug interactions (GDIs). Results from previous studies on ginsenosides' effects on human drug-metabolizing P450 enzymes are inconsistent and confusing. Herein, we first evaluated the inhibitory effects of fifteen ginsenosides and sapogenins on human CYP1A2, CYP2C9, CYP2C19, CYP2D6 and CYP3A4 enzymes by using commercially available fluorescent probes. The structure-activity relationship of their effects on the P450s was also explored and a pharmacophore model was established for CYP3A4. Moreover, substrate-dependent phenomena were found in ginsenosides' effects on CYP3A4 when another fluorescent probe was used, and were further confirmed in tests with conventional drug probes and human liver microsomes. These substrate-dependent effects of the ginsenosides may provide an explanation for the inconsistent results obtained in previous GDI reports.

## Introduction

**Figure 1:**
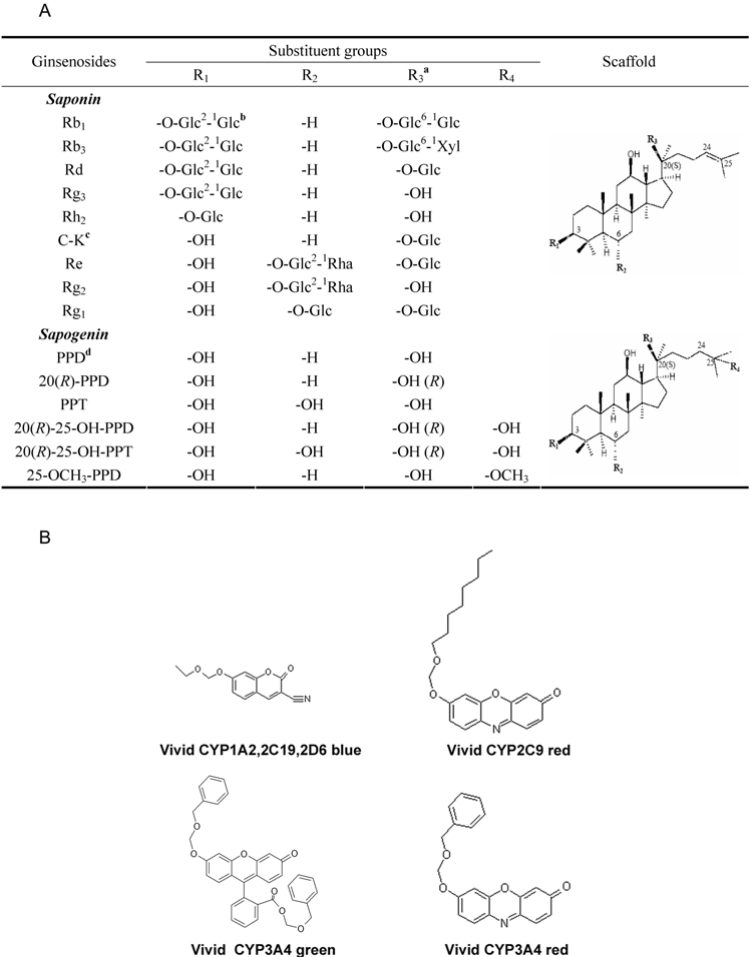
Structures of ginsenosides (A), and Vivid® fluorescent probes (B). Note: *a*. The C-20 configurations of the test ginsenosides are 20(*S*) except when indicated behind the substituent groups in this column. *b*. Glc: β-D-glucopyranosyl; Xyl: β-D-xylopyranosyl; Rha: α-L-rhamnopyranosyl. Numerical superscripts indicate the carbons at glucosidic bonds. *c*. C-K: ginsenoside Compound K. *d*. PPD: protopanaxadiol; PPT: protopanaxatriol.

With the increasing use of alternative medicine and a wide spread of combination therapies for various diseases, there is an increasing interest in determining drug-drug, drug-nutrient, and drug-dietary supplements interactions. For example, *Panax ginseng*, a traditional herbal medicine used in the Eastern Asia for more than 2000 years [1], is presently being used worldwide as one of the most common complementary alternative medicines. Ginseng, the root of *Panax ginseng*, has diverse pharmacological activities, including effects on the central nervous system, antineoplastic and immunomodulatory effects [2]. In the clinical settings, however, co-administration of ginseng or its extracts with other therapeutic agents (e.g. warfarin, digoxin and phenelzine) may lead to ginseng-drug interactions (GDIs) [3], [4]. Ginsenosides (Fig. 1a), the major active components of ginseng, may account for the GDIs and other adverse effects [5]. It is postulated that most metabolic drug-drug interactions can be attributed to inhibition or induction of drug-metabolizing cytochrome P450 (CYP or P450) enzymes [6]. Therefore, we hypothesized that investigations on the effects of ginsenosides on P450s will help elucidate the mechanism of GDIs.

Unfortunately, the reported effects of ginseng extracts or ginsenosides on P450s are inconsistent, even confusing. For instance, ginsenoside Rd weakly inhibits CYP3A4 and CYP2D6 and inhibit CYP2C19 and CYP2C9 to an even lesser extent; and ginsenosides Rb_1_, Rb_2_, Re, and Rg_1_ do not significantly affect CYP1A2, CYP2C9, CYP2C19, CYP2D6 or CYP3A4 [7]. In another study, however, ginsenosides Rd and Rb_2_ inhibited CYP2C19-dependent S-mephenytoin 4′-hydroxylation and Rd inhibited CYP2D6-mediated bufuralol 1′-hydroxylation [8]. Moreover, standardized *Panax ginseng* and *Panax quinquefolius* extracts decrease the 7-ethoxyresorufin O-dealkylation activities of human CYP1A1, CYP1A2, and CYP1B1, but ginsenosides Rb_1_, Rb_2_, Rc, Rd, Re, Rf or Rg_1_ have no significant effects [9]. Additionally, GDIs are probably mediated by ginsenoside metabolites (e.g. protopanaxadiol and protopanaxatriol) rather than natural ginsenosides [10], since the ginsenosides are deglycosylated by enterobacteria before they enter the circulation [11]. To date, some 80 ginsenosides have been isolated from *Panax* species [12] and new ginsenosides are being found. Recently, we identified and characterized two novel potent antitumor ginsenosides, 20(*R*)-dammarane-3*β*, 12*β*, 20, 25-tetrol (25-OH-PPD) [13] and 20(*S*)-25-methoxyl-dammarane-3*β*, 12*β*, 20-triol (25-OCH_3_-PPD) [14]; their effects on P450s are still unknown.

We also speculated that there are structure-activity relationships (SAR) for the effects of ginsenosides on P450s. Although several ligand-based and structure-based models have been established for substrates and/or inhibitors of P450s [15], the SAR of the effects of the ginsenosides on P450s were rarely addressed. In the present study, we systematically evaluated the effects of 15 ginsenosides and sapogenins (Fig. 1A) on five major human drug-metabolizing P450 enzymes with commercially available Vivid® substrates to use as probes (Fig. 1B). Subsequently, the SAR for these effects was explored to generate new knowledge about ginsenoside-P450 interactions at the molecular level. Moreover, we also selected different fluorescent and conventional probes to determine whether the effects of different ginsenosides on CYP3A4, a key P450 enzyme with the largest substrate repository, are substrate- and analog-dependent.

## Results

### Ginsenosides and sapogenins affect P450 enzymes in an analog- and enzyme-dependent manner

The parameters used to determine the activities of the P450 enzymes are summarized in Table 1. Reported potent inhibitors of respective P450 enzyme were evaluated to validate the efficiency of these fluorescent assays (Table 2). These inhibitors show comparable activities against respective P450s to the documented reports [16]–[19]. Additionally, the inhibitory effects of methanol, a vehicle used in the study, were carefully evaluated. Methanol at the final concentration of 1% had no significant inhibitory effect against Vivid® blue metabolism by CYP1A2 or green metabolism by CYP3A4, and had minimal inhibition against Vivid® blue metabolism by CYP2C19 (Table 2). However, 1% methanol significantly inhibited Vivid® CYP2C9 red metabolism (by 42.4%) and blue metabolism by CYP2D6 (27.5%). At the final concentration of methanol of 0.5% or 0.1%, the inhibition against substrate metabolism by methanol was all either negligible or within an acceptable range (Table 2).

**Table 1:** Parameters of the enzymatic reactions used to determine the activities of P450 enzymes.

Vivid® assay	Substrate concentration (µM)	Wavelengths (nm)		CYP450 concentration (nM)	Linear range of RFU∼t curve (min)**^a^**
		Excitation	emission		
CYP1A2 blue	3	409	460	5	0∼15
CYP2C9 red	2	530	585	10	0∼30
CYP2C19 blue	10	409	460	5	0∼15
CYP2D6 blue	10	409	460	10	0∼30
CYP3A4 green	2	485	530	5	2∼12
CYP3A4 red	3	530	585	5	0∼10

**Note:** ***a***. The linear range was determined by visual inspection; parameters for substrate concentration, wavelength and CYP450 concentration were provided by the kit manufacturer.

**Table 2:** IC_50_ values of the ginsenosides and sapogenins against P450 enzymes.

Test compounds	IC_50_ (µM) (%inhibition at 50 µM) **^a^**					
	CYP1A2 blue	CYP2C9 red**^b^**	CYP2C19 blue	CYP2D6 blue	CYP3A4 green	CYP3A4 red
Positive control **^c^**	1.5	0.50	0.25	0.0076	0.49	0.97
***Saponin***						
Rb_1_	37.8	>50 (25.2%)	>100 (20.6%)	>50 (29.1%)	>100 (10.1%)	>100 (4.4%)
Rb_3_	>100 (31.3%)	>50 (17.6%)	>100 (5.0%)	>50 (49.4%)	99.7	68.8
Rd	>100 (38.3%)	>50 (34.8%)	>100 (27.7%)	>50 (18.1%)	>100 (12.6%)	72.7
Rg_3_	32.4	18.5	47.0	9.3	86.4	25.2
Rh_2_	>100 (39.4%)	30.9	49.9	>50 (20.3%)	47.0	9.8
C-K	23.8	9.1	45.9	>50 (49.4%)	30.5	9.1
Re	>100 (28.1%)	>50 (38.0%)	>100 (8.8%)	>50 (44.2%)	>100 (4.3%)	66.1
Rg_2_	>100 (29.2%)	>50 (20.1%)	>100 (14.8%)	>50 (37.2%)	>100 (19.4%)	47.0
Rg_1_	>100 (30.2%)	>50 (21.7%)	>100 (11.7%)	>50 (17.7%)	>100 (8.1%)	>100(16.9%)
***Sapogenin***						
PPD	86.6	8.0	52.6	>50 (25.9%)	9.3	43.1
20(*R*)-PPD	>100 (17.1%)	6.7	19.2	>50 (34.9%)	10.3	58.6
PPT	>100 (13.1%)	31.6	46.9	>50 (18.9%)	7.4	>100(39.4%)
20(*R*)-25-OH-PPD	>100 (22.8%)	7.6	69.1	>50 (31.8%)	25.6	A.A. **^d^**
20(*R*)-25-OH-PPT	>100 (16.9%)	8.5	43.9	>50 (10.0%)	27.2	A.A.
25-OCH_3_-PPD	>100 (4.8%)	7.5	26.5	>50 (12.6%)	17.7	>100(25.4%)

**Note:** ***a***. The percent inhibition of ginsenosides against the respective P450 enzymes is shown when its IC_50_ value is greater than the maximum concentration assayed.

***b***. The maximum concentration of ginsenosides evaluated for their effects on CYP2C9 and CYP2D6 were 50 µM due to the marked solvent effect of 1% methanol on these two P450 enzymes (inhibition by 42.4% and 27.5%, respectively). When the final concentration of methanol was decreased to 0.5%, the solvent effects were acceptable for these two enzymes (10.1% and 18.9%, respectively). 1% methanol had no inhibition against CYP1A2 and CYP3A4 and had an acceptable inhibitory effect on CYP2C19 (9.7%).

***c***. Positive control compounds were α-naphthoflavone (for CYP1A2), sulfaphenazole (CYP2C9), miconazole nitrate salt (CYP2C19), quinidine (CYP2D6), and ketoconazole (CYP3A4), respectively.

***d***. A.A. = apparent activation. 100 µM 25-OH-PPD and 25-OH-PPT increased the turnover of Vivid® CYP3A4 red by more than 100%.

Subsequently, we determined the effects of the ginsenosides and sapogenins on five major cDNA-expressed P450 enzymes. The compounds had weak inhibitory effects against CYP1A2, with the exception of Rb_1_, Rg_3_, and Compound K (C-K) which had moderate inhibitory effects with IC_50_ values less than 50 µM (Table 2). Ginsenosides Rg_3_, Rh_2_, and C-K and all sapogenins exhibited moderate inhibition against CYP2C19, while they more potently inhibited CYP2C9 and CYP3A4 (green substrate) (p<0.05). Rg_3_ exerted relatively weak inhibitory effects against Vivid® CYP3A4 green metabolism (Table 2). However, it potently inhibited CYP2D6, for which all of the other ginseng compounds had IC_50_ values greater than 50 µM. Ginsenosides Rb_1_, Rb_3_, Rd, Re, Rg_2_ and Rg_1_ weakly affected all five P450 enzymes, with the exception of Rb_1_ and C-K, which moderately inhibited CYP1A2 (Table 2). All of the sapogenins had weak inhibitory effects on CYP1A2 and CYP2D6 (Table 2).

### There is a SAR in the effects of ginsenosides on P450s

Considering that the same core structure shared by these ginsenosides, the location, number and type of the glycosyl groups covalently linked to the dammarane scaffold may account for the their varying effects on P450 enzymes (Fig. 1A and Table 2). For CYP2C9 and CYP2C19, the inhibition patterns of ginsenosides are similar. Ginsenosides that do not have a glycosyl group on R_2_ and have two or fewer glycosyl groups on R_1_ and R_3_ as well as sapogenins generally have moderate to potent effects on CYP2C9 and CYP2C19. As for CYP3A4 (green substrate), the inhibitory activities of the ginsenosides and sapogenins decreased as the number of glycosyl groups increased.

### 3D-SAR is shown in the effects of ginsenosides on Vivid® CYP3A4 green metabolism

**Figure 2:**
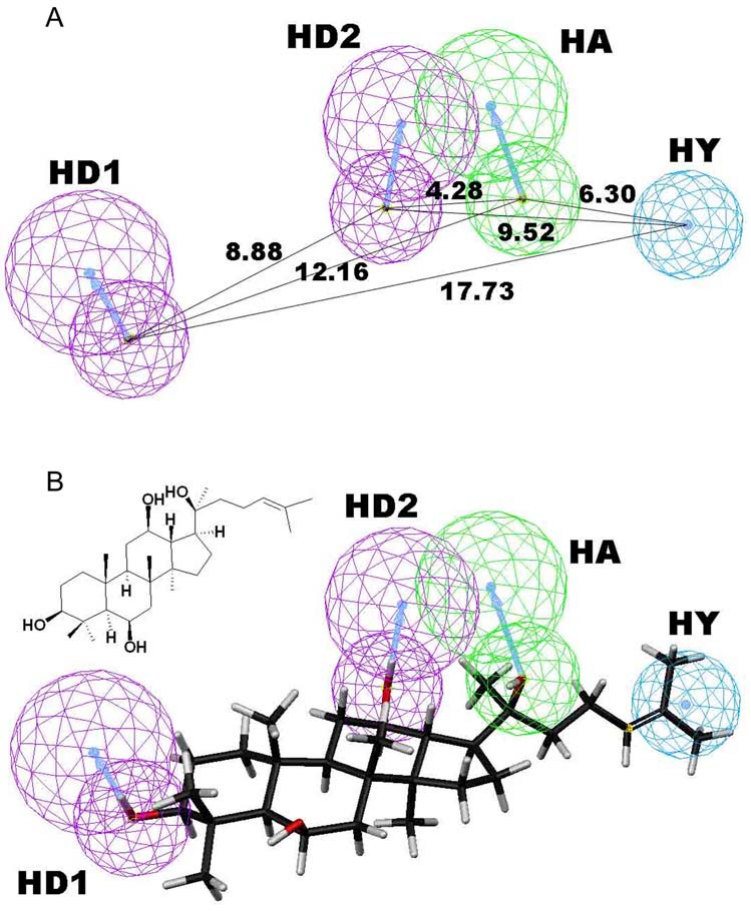
Shape-based pharmacophore model of the inhibition of Vivid® CYP3A4 green activity by ginsenosides. The model was generated from the 3D structure of PPT and its activities against CYP3A4. PPT fitted into the model was shown in the figure. Blue represents the shape space, black represents carbon atoms, red represents oxygen atoms, and white represents hydrogen atoms.

We applied the HipHop process from Catalyst software, which compares diverse and highly active compounds to derive 3D hypotheses based on common chemical features, without considering biological activities. The final training set consisting of 6 highly active compounds was submitted for pharmacophore building. The best hypothesis (Hypo1), consisting of four features, namely, one hydrogen-bond acceptor, one hydrophobic point, and two hydrogen-bond donors (Fig. 2A). A representation of the chemical features mapped onto representative studied compound (PPT) is depicted in Fig. 2B. In this model, the hydrogen bond acceptor seems to be mapped to the oxygen atom substituted at atom C20, and the hydrophobic group fit well with the aliphatic chain attached as side-chain to this moiety. The analysis shows that hydroxyl substitutions at atom C3 and C12 are crucial. Hydrogen-bond donors at these atomic positions will increase the bioactivity. From Fig. 2B, it is evident that the hydroxyl group substituted at atom C3 and C12 serves to be hydrogen-bond donors (HD1 and HD2). Thus replacement with other substituent group at oxygen atoms of C3-OH or C12-OH will result in decreased potency.

### The effects of the ginsenosides on CYP3A4 were substrate-dependent

**Figure 3:**
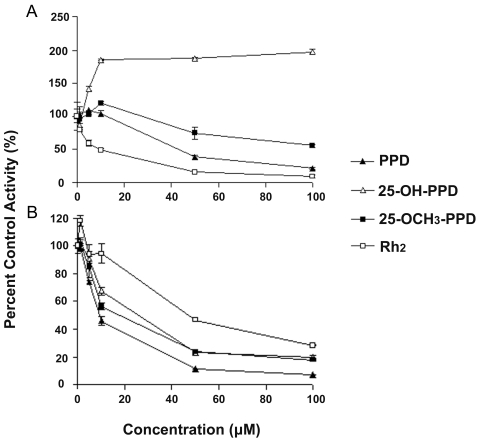
Effects of four different ginsenosides on the Vivid® CYP3A4 red assay (A) and green assay (B). Each point is the mean value of triplicate samples, with error bars representing RSD values.

Considering potential influences of substrate selection on the evaluation of the effects of the ginsenosides on the catalytic activity of CYP3A4, another fluorescent probe, Vivid® CYP3A4 red (Fig. 1B), was used. The assay conditions are also summarized in Table 1. The solvent effect of methanol (1%) on CYP3A4 red metabolism was negligible. The IC_50_ value of ketoconazole on the CYP3A4 red assay was 0.97 µM (Table 2). As for the ginsenosides and sapogenins, the inhibition profiles using the red substrate and green substrate were not consistent. For the same saponin, the inhibition potency determined using the red substrate was greater than using the green substrate (Table 2) (p<0.05). However, the pattern was the opposite for sapogenins: the inhibition potency was overestimated using the green substrate compared to the red substrate (Table 2). Strikingly, 25-OH-PPD and 25-OH-PPT activated the catalytic activity of CYP3A4 when using red substrate. The representative influence profiles of PPD, 25-OH-PPD, 25-OCH_3_-PPD and Rh_2_ on CYP3A4 activities using either red or green substrate are shown in Fig. 3. In the Vivid® red assay (Fig. 3A), 25-OH-PPD potently activated CYP3A4 (about two-fold) at a low concentration (10 µM). It was notable that at higher concentrations 25-OH-PPD activated CYP3A4 red metabolism in a saturated profile. At 10 µM, 25-OCH_3_-PPD weakly activated CYP3A4 (about 20%), but weakly inhibited the enzyme at high concentrations. PPD and Rh_2_ inhibited CYP3A4 in a dose-dependent manner. However, the effect of Rh_2_ (IC_50_ = 9.8 µM) was more potent than PPD (IC_50_ = 43.1 µM) (p<0.01). In the Vivid® green assay (Fig. 3B), however, all four tested compounds inhibited CYP3A4 in a dose-dependent manner, with the potency decreasing in the order: PPD>25-OCH_3_-PPD>25-OH-PPD>Rh_2_.

The effects of the ginsenosides and sapogenins on CYP3A4 were also substrate-dependent when using human liver microsomes and different conventional probes. CYP3A4-catalyzed reactions (carbamazepine 10,11-epoxidation and nifedipine oxidation) were used to evaluate the potential ginsenoside-drug interactions. We employed high performance liquid chromatography/tandem mass spectrometry (HPLC-MS-MS) analysis to detect the reaction products carbamazepine 10,11-epoxide (CBZ-E) and oxidized nifedipine (ONF) of respective drug substrates carbamazepine (CBZ) and nifedipine (NF).

**Figure 4:**
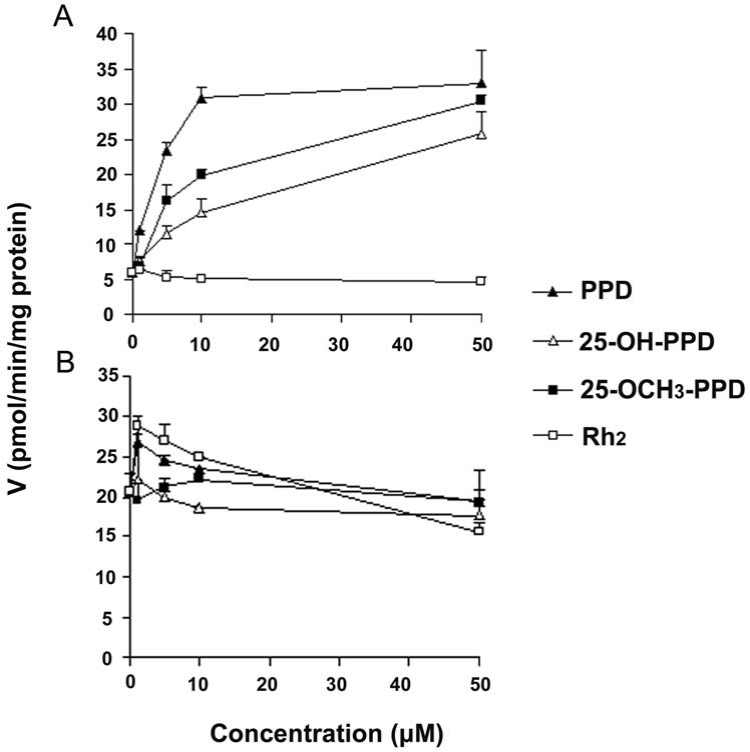
Effects of four different ginsenosides on the formation of carbamazepine 10,11-epoxide (A) and oxidized nifedipine (B). Data are avereages of triplicate samples.

Strikingly, PPD, 25-OH-PPD and 25-OCH_3_-PPD substantially activated *in vitro* CBZ metabolism in a dose-dependent manner, with the potency as follows: PPD>25-OCH_3_-PPD>25-OH-PPD (Fig. 4A). It is notable that PPD potently activated (about five-fold) the CBZ metabolism, even at the low concentration (10 µM), and the activation by PPD at high concentrations reached saturation. However, Rh_2_ did not significantly alter CBZ metabolism (Fig. 4A). Reported activator α-naphthoflavone (10 µM) potently activated CBZ metabolism (increased by 772%), whereas ketoconazole (10 µM) exerted a substantial inhibitory effect (decreased metabolism by 85.9%) compared to the vehicle control.

As for NF *in vitro* metabolism, Rh_2_ and PPD exerted a weak activation effect at low concentrations (1∼10 µM). However, the high concentration (50 µM) of Rh_2_ weakly inhibited CYP3A4-catalyzed NF oxidation. The effects of 25-OCH_3_-PPD and 25-OH-PPD on NF metabolism were negligible (Fig. 4B). α-Naphthoflavone (10 µM) did not significantly alter *in vitro* NF metabolism, whereas ketoconazole (10 µM) inhibited CYP3A4-catalyzed NF metabolism almost completely (decreased by 94.9%), compared to the vehicle control.

## Discussion

Potential GDIs in the clinical settings make it necessary to investigate the influences of major ginseng components on drug-metabolizing enzymes. To that end, we studied the effects of 15 ginsenosides and sapogenins on P450-mediated drug metabolism. Compared to microsomal assays with conventional probe drugs using HPLC analysis, fluorescent methods for determining P450 activity are more high-throughput and reproducible [20]–[24] and have been extensively employed in screening for inhibitors and inducers of P450s [24]–[27]. Using the fluorescent probes, we found that ginsenoside Rb_1_, Re and Rg_1_ had no significant effects on major P450s, with the exception that Rb_1_ moderately inhibited CYP1A2 in our assay. A previous report [28] indicated that ginsenoside Rd inhibits CYP3A4-mediated testosterone 6β-hydroxylation with an IC_50_ value of 62 µM, however, we did not observe any significant influence of Rd on any of the P450s tested. More recently, Liu et al. found that ginsenoside metabolites (C-K, PPD and PPT) are more potent P450 inhibitors than natural ginsenosides [10]. In our present study, we also found that sapogenins are more potent than saponins in their effects on CYP3A4, despite the fact that the IC_50_ values we obtained for prosapogenin C-K and Rh_2_ were lower. It is noteworthy, however, that several saponins in our assay were found to have activities comparable to (Rg_3_, Rh_2_ and C-K against CYP2C9 and CYP2C19) or even greater than (Rb_1_, Rg_3_ and C-K against CYP1A2, and Rg_3_ against CYP2D6) sapogenins (p<0.05). These findings indicate that natural ginsenosides still have the potential to inhibit certain P450 enzymes and possibly lead to GDIs *in vivo*. Information about the circulating levels of these ginsenosides and sapogenins in humans would be helpful to determine whether these interactions occur *in vivo*. However, except for only a few ginsenosides (Rb_1_, Rg_1_ [29] and Rg_3_ [30]), such information is not available in the literature due to limited pharmacokinetic studies of ginsenosides in human.

Our data showed that a SAR exists in the effects of ginsenosides on almost all of the P450 enzymes examined, especially on CYP2C9, CYP2C19 and CYP3A4 (green substrate) (Table 2). Apparently, the similarities and differences among the activities of tested ginsenosides against each of the P450s are consistent with the fact that these compounds are analogs and differ in only four substituent groups. Furthermore, a pharmacophore (Fig. 2) was established based on the data set from the CYP3A4 green assay to explore more information about effects of ginsenosides and sapogenins on this key enzyme, which participates in the metabolism of more than half of pharmaceuticals. The model shows that hydroxyl substitutions at atom C3 and C12 play a key role in the inhibitory potency of ginsenosides and sapogenins against Vivid® CYP3A4 green metabolism. Presence of hydrogen-bond donors at these atomic positions will increase the bioactivities of ginsenosides and sapogenins on CYP3A4. Therefore, substitution with glycosyl group(s) at oxygen atoms of C3-OH will result in decreased potency of the compounds.

The inconsistencies existing in the previous reports (e.g. effects of Rd and Rb_2_ on CYP2C19 [7], [8]) and conflicts between our data and previous reports (e.g. effects of saponins on several P450s [10]) on the inhibition of P450s by ginsenosides can most likely be attributed to the different substrates and assay systems used to determine the P450 activities. It is recommended that multiple probes should be used when P450-mediated drug interactions are evaluated [24]. This is especially the case for CYP3A4, because its drug interaction patterns were substrate-dependent [31]. We therefore evaluated the effects of the ginsenosides and sapogenins on the activity of human CYP3A4 by using another fluorescent probe and two conventional drug substrates. Interestingly, the influence patterns of these compounds against CYP3A4 using the red substrate were distinct from those observed using the green substrate. In subsequent microsomal studies with CBZ and NF as substrates, these substrate-dependent and analog-dependent phenomena were further validated. CBZ *in vitro* metabolism was potently activated by PPD, 25-OCH_3_-PPD and 25-OH-PPD, even at low concentrations (5∼10 µM). CBZ is an anticonvulsant agent used in the treatment of epilepsy, acute mania and bipolar disorder [32]. Due to co-administration of ginseng products among some epilepsy patients [33], ginseng or ginseng-based products have the potential to promote the metabolism of CBZ, and thus decrease the bioavailability of this drug. However, whether PPD, 25-OCH_3_-PPD, or 25-OH-PPD can activate CBZ *in vivo* metabolism needs further investigation.

The substrate-dependent phenomena and atypical kinetics of P450-mediated drug-drug interactions, especially for CYP3A4, were documented in several previous *in vitro* studies [31], [34], [35]. There have also been several reports concerning the activation of P450s. Tea and tea polyphenols were found to heteroactivate CYP1A1 with a fluorescent probe [36]. Endogenous steroids were reported to activate CYP3A4-mediated CBZ 10,11-epoxidation [37], [38]. As for ginsenosides, Rc was documented to activate CYP2C9 activity, while Rf activated CYP3A4 in fluorescent assays [7]. These substrate-dependent activation events may be due to the large active site of P450s, especially CYP3A4, as evidenced by its crystal structure [39]. CYP3A4 can bind multiple ligands simultaneously, a property that may contribute to its complex cooperative effects [40]–[43]. Thus, different inhibition profiles of the test compounds against CYP3A4 result when probes with different structures are used. As to the mechanism of heteroactivation of CBZ metabolism by endogenous steroids, CBZ may be more easily catalyzed due to direct interaction with steroids in the active site, which may change the activity of CYP3A4 [44]. Interestingly, ginsenoside sapogenins that have stimulatory effects on CBZ metabolism (PPD, 25-OCH_3_-PPD and 25-OH-PPD) have structures with similarity to steroids, and may activate CYP3A4 activity by interacting with CBZ in the active site. However, saponin ginsenosides (such as Rh_2_), which are of relatively large size, may hinder the co-binding events and consequently lead to no or inhibitory effects on CYP3A4. Nevertheless, the molecular and structural basis of these substrate- and analog-dependent effects is still not clear and needs further investigation.

Our observations of substrate-dependent effects of ginsenosides and sapogenins on CYP3A4 suggest that GDIs may also be substrate-dependent. In contrast with reports mentioned in the ‘Introduction’ section, several *in vivo* studies suggested that ginseng or ginseng extracts have little effect on P450 activities. Coadministration of ginseng did not alter the pharmacokinetics and pharmacodynamics of warfarin in human subjects [45]. Gurley et al found that supplementation of *Panax ginseng* did not have any significant effects on CYP1A2, 2D6, 2E1 or CYP3A4 activities *in vivo* when their activity was examined by use of probe-drug cocktails [46]. Our studies may provide an explanation for the inconsistencies in clinical GDI reports. Inhibition or activation, and the extent of these effects of ginseng or ginseng products on P450s, are relative and based on the substrate(s) selected in the assay. Translation from data obtained with only one ‘probe’ to other drugs metabolized by the same P450 enzyme will probably lead to *ex parte* conclusion on GDI, and may lead researchers to overlook potential interactions of ginseng with drugs other than the probe. Therefore, multiple probe drugs are needed for careful *in vitro* and *in vivo* evaluation of P450-mediated GDIs, and drug-drug interactions to a larger extent, especially in case of CYP3A4.

In summary, we evaluated the *in vitro* effects and the SAR of fifteen ginsenosides and sapogenins on five major human drug-metabolizing P450 enzymes. In addition, we also found that substrate-dependent phenomena exist in the effects of the ginsenosides and sapogenins when employing different fluorescent and conventional probes. To the best of our knowledge, this study is the first report on the SAR and substrate-dependence of the effects of ginsenosides on P450s. The information derived will enhance our understanding of GDIs, and provide a possible explanation for the inconsistent results obtained in previous reports.

## Materials and Methods

### Chemicals and Reagents

All ginsenosides and sapogenins, isolated as described previously [13], [14], were at least 95% pure. α-naphthoflavone, miconazole nitrate salt, quinidine, ketoconazole, carbamazepine (CBZ), carbamazepine 10,11-epoxide (CBZ-E), nifedipine (NF), oxidized nifedipine (ONF) and ammonium acetate were purchased from Sigma-Aldrich (St. Louis, MO, USA). Sulfaphenazole was a generous gift from Ms. Yuanyuan Dai at Cancer Hospital/Institute, Chinese Academy of Medical Sciences (Beijing, China). Na_3_PO_4_ was purchased from Sinopharm Chemical Reagent Co., Ltd. (Shanghai, China). Pooled human liver microsomes (HLM), Solution A (20×, containing 1.3 mM NADP^+^, 3.3 mM glucose-6-phosphate, 3.3 mM MgCl_2_) and Solution B (100×, 0.4 U/ml glucose-6-phosphate dehydrogenase) of the NADPH regenerating system were supplied by BD Biosciences (San Jose, CA, USA). Chromatographic grade methanol and acetonitrile were purchased from Tedia Company (Fairfield, OH, USA).

### Vivid® P450 assays

The inhibition of the catalytic activities of cDNA-expressed human P450 enzymes by ginsenosides was determined using Vivid® P450 screening kits (CYP1A2 blue, CYP2C9 red, CYP2C19 blue, CYP2D6 blue, CYP3A4 green and red) according to the manufacturer's instructions (Invitrogen Corporation; Carlsbad, CA, USA). Each kit contained P450 reaction buffer, P450 BACULOSOMES® reagent, fluorescent substrate, fluorescent standard, the regeneration system (333 mM Glucose-6-phosphate and 30 U/ml glucose-6-phosphate dehydrogenase in 100 mM potassium phosphate pH 8.0), and 10 mM NADP^+^ in 100 mM potassium phosphate, pH 8.0.

In brief, the assays were carried out in Costar® black-wall 96-well plates with ultra thin clear bottoms (Corning Inc, Corning, NY, USA) in a kinetic assay mode. Stock solutions (10 mM) of ginsenosides in methanol were prepared and diluted to various concentrations (2.5× 100, 50, 10, 5, 1 µM). For each well, 40 µL of solution of test compounds or vehicle was incubated with 50 µL pre-mixture (mixture of BACULOSOMES® reagent, regeneration system and reaction buffer) at 37°C for 20 min. The reaction was initiated by adding 10 µL of a mixture of substrate and NADP^+^ per well with a respective concentration of Vivid® substrate (Table 1). The plate was read immediately for fluorescence changes every 0.5 min at 37°C for 30 min using a FlexStation II384 fluorometric plate reader (Molecular Devices, Sunnyvale, CA, USA) with respective excitation and emission wavelengths for each P450 enzyme (Table 1). The final methanol volume in the reaction was less than or equal to 1%. Inhibitory potencies of test compounds (IC_50_ values) were compared by using Student' t-test. Difference was considered to be significant when the two-tailed p-value was less than 0.05.

### Pharmacophore generation

The compounds were built using Catalyst (Accelrys corporation, San Diego, CA, USA) 2D–3D sketcher, and a family of representative conformations was generated for each ginsenoside or sapogenin using the best conformational analysis method. A maximum number of 250 conformations of each compound were selected using “best conformer generation” option with a constraint of 20 kcal/mol energy thresholds above the global energy minimum to ensure maximum coverage of the conformational space. Based on the conformations for each compound, Catalyst 4.10 software package was employed to construct possible pharmacophore models. When generating a hypothesis, catalyst attempts to minimize a cost function consisting of two terms. Analysis of functional groups on each compound in the training set revealed that three chemical features, hydrogen-bond acceptor (HA), hydrogen-bond donor (HD), and hydrophobic group (HY), could effectively map all of the critical chemical features. Hence, the three features were selected to form the essential information in this hypothesis generation process. The best predictive hypothesis (Hypo1), produced by HipHop process encoded in Catalyst 4.10, has four features: one hydrogen-bond acceptor, one hydrophobic point, and two hydrogen-bond donor, which was characterized by the highest cost difference, the lowest rms divergence, and the best correlation coefficient. Remarkably, the highest active compound (PPT) can be nicely mapped onto the Hypo1 model by the best fit values.

### Microsomal studies

The effects of the ginsenosides and sapogenins on the *in vitro* metabolism of CBZ were evaluated using HLM. Triplicate microsomes (50 µL, 20 mg/ml) were treated sequentially with 20 µL 1 mM CBZ 10% DMSO (v/v) aqueous solution, 20 µL aqueous solutions of test ginsenosides containing 25% methanol (v/v) at different concentrations (1, 5, 10, and 50 µM) or α-naphthoflavone (10 µM) or ketoconazole (10 µM), and 850 µL 0.1 M Na_3_PO_4_ solution, and pre-incubated at 37°C for 5 min. Vehicle control samples were treated with methanol at a final concentration of 0.5% instead of test compounds. The reaction was initiated by adding 60 µL pre-warmed NADPH-regenerating system (50 µL BD solution A and 10 µL BD solution B). After incubation at 37°C for 30 min, the reaction system was quenched by adding 0.4 ml ice-cold acetonitrile, which was mixed well and centrifuged at 4°C (10,000 g, 10 min). The supernatant (0.4 ml) was collected, mixed with 0.8 ml acetonitrile and then centrifuged at 4°C (10,000 g, 10 min). The supernatant (30 µL) was diluted with 970 µL ddH_2_O and 50 µL of the mixture was injected onto the high performance liquid chromatography/tandem mass spectrometry (HPLC-MS-MS) system for analysis. The calibration samples were prepared with standard solutions of CBZ-E (0.00457, 0.0137, 0.0412, 0.123, 0.370, 1.11, and 3.33 µM) and processed identically to the samples before analysis, except that solution A was added after microsomal proteins were precipitated by acetonitrile. Microsomal studies using NF as the substrate were conducted as described above for CBZ with several modifications. The NF concentration was 1 µM. The supernatant (0.4 ml) of the reaction system after quenching and centrifugation was also combined with 0.8 ml acetonitrile and centrifuged, and 10 µL of the resultant supernatant was directly injected onto the HPLC-MS-MS system for analysis. The calibration standards for ONF included 0.4572, 1.372, 4.115, 12.35, 37.04, 111.1, 333.3, and 1000 nM.

### LC-MS-MS analysis

The HPLC was performed using an Agilent 1200 system (Palo Alto, CA, USA) equipped with a binary pump and an auto-sampler. For CBZ-E analysis, an Agilent Zorbax SB-C18 column (4.6×150 mm, 5 µm) was used, and the mobile phase consisted of 50% eluent A (aqueous solution containing 0.5% acetic acid) and 50% eluent B (acetonitrile) at the flow rate of 0.4 ml/min. The elution lasted for 8 min. For ONF analysis, an Agilent Zorbax Eclipse® XDB-C8 column (4.6×150 mm, 5 µm) was used; the mobile phase consisted of eluent A (10 mM ammonium acetate) and eluent B (acetonitrile) at the flow rate of 0.6 ml/min. The gradient elution program was as follows: started with 70% B for 1.5 min; changed to 95% B within 1 min; held at 95% B for 5.5 min; returned rapidly to 70% B; and held at 70% B for 2 min.

The MS analysis was performed on a 4000Q Trap™ triple quadrupole mass spectrometer (Applied Biosystems/MDS Sciex Instruments, Concord, Ontario, Canada) equipped with a Turbo V ion source (TurboIonSpray™ probe was used), a Dell™ computer, and Analyst™ software. Electrospray ionization in the positive mode was employed to analyze CBZ-E and ONF, respectively. The compound parameters for CBZ-E and ONF were: declustering potential, 65 and 95 units, and collision energy, 39 and 40 units, respectively. The ion source parameters were as follows: curtain gas, 15 units; collision gas, medium; ion spray voltage, 4000 and 5000 V, respectively; temperature, 650 and 350°C, respectively; ion source gas 1, 60.0 and 65.0 units, respectively; ion source gas 2, 45.0 units. The fragmentation transitions for multiple reaction monitoring (MRM) were *m/z* 253.1→180.2 for CBZ-E and *m/z* 345.1→284.3 for ONF. The representative calibration curve for CBZ-E is: *Y* = 147*X*+358 (r = 0.9993) with 1/*X^2^* weighting, and the representative calibration curve for ONF is: *Y* = 3570*X*−2410 (r = 0.9976) with 1/*X* weighting, where *Y* represents the peak area of the analyte, and *X* represents the analyte concentration.

## References

[1] Helms S (2004). Cancer prevention and therapeutics: Panax ginseng.. Altern Med Rev.

[2] Attele AS, Wu JA, Yuan CS (1999). Ginseng pharmacology: multiple constituents and multiple actions.. Biochem Pharmacol.

[3] Fugh-Berman A (2000). Herb-drug interactions.. Lancet.

[4] Izzo AA (2005). Herb-drug interactions: an overview of the clinical evidence.. Fundam Clin Pharmacol.

[5] Sparreboom A, Cox MC, Acharya MR, Figg WD (2004). Herbal remedies in the United States: potential adverse interactions with anticancer agents.. J Clin Oncol.

[6] Wienkers LC, Heath TG (2005). Predicting in vivo drug interactions from in vitro drug discovery data.. Nat Rev Drug Discov.

[7] Henderson GL, Harkey MR, Gershwin ME, Hackman RM, Stern JS (1999). Effects of ginseng components on c-DNA-expressed cytochrome P450 enzyme catalytic activity.. Life Sci.

[8] He N, Xie HG, Collins X, Edeki T, Yan Z (2006). Effects of individual ginsenosides, ginkgolides and flavonoids on CYP2C19 and CYP2D6 activity in human liver microsomes.. Clin Exp Pharmacol Physiol.

[9] Chang TK, Chen J, Benetton SA (2002). In vitro effect of standardized ginseng extracts and individual ginsenosides on the catalytic activity of human CYP1A1, CYP1A2, and CYP1B1.. Drug Metab Dispos.

[10] Liu Y, Zhang JW, Li W, Ma H, Sun J (2006). Ginsenoside metabolites, rather than naturally occurring ginsenosides, lead to inhibition of human cytochrome P450 enzymes.. Toxicol Sci.

[11] Hasegawa H (2004). Proof of the mysterious efficacy of ginseng: basic and clinical trials: metabolic activation of ginsenoside: deglycosylation by intestinal bacteria and esterification with fatty acid.. J Pharmacol Sci.

[12] Fuzzati N (2004). Analysis methods of ginsenosides.. J Chromatogr B Analyt Technol Biomed Life Sci.

[13] Wang W, Zhao Y, Rayburn ER, Hill DL, Wang H (2007). In vitro anti-cancer activity and structure-activity relationships of natural products isolated from fruits of Panax ginseng.. Cancer Chemother Pharmacol.

[14] Zhao Y, Wang W, Han L, Rayburn ER, Hill DL (2007). Isolation, structural determination, and evaluation of the biological activity of 20(S)-25-methoxyl-dammarane-3beta, 12beta, 20-triol [20(S)-25-OCH3-PPD], a novel natural product from Panax notoginseng.. Med Chem.

[15] de Groot MJ (2006). Designing better drugs: predicting cytochrome P450 metabolism.. Drug Discov Today.

[16] Weaver R, Graham KS, Beattie IG, Riley RJ (2003). Cytochrome P450 inhibition using recombinant proteins and mass spectrometry/multiple reaction monitoring technology in a cassette incubation.. Drug Metab Dispos.

[17] Brown HS, Galetin A, Hallifax D, Houston JB (2006). Prediction of in vivo drug-drug interactions from in vitro data: factors affecting prototypic drug-drug interactions involving CYP2C9, CYP2D6 and CYP3A4.. Clin Pharmacokinet.

[18] Niwa T, Shiraga T, Takagi A (2005). Effect of antifungal drugs on cytochrome P450 (CYP) 2C9, CYP2C19, and CYP3A4 activities in human liver microsomes.. Biol Pharm Bull.

[19] Kim MJ, Kim H, Cha IJ, Park JS, Shon JH (2005). High-throughput screening of inhibitory potential of nine cytochrome P450 enzymes in vitro using liquid chromatography/tandem mass spectrometry.. Rapid Commun Mass Spectrom.

[20] Trubetskoy OV, Gibson JR, Marks BD (2005). Highly miniaturized formats for in vitro drug metabolism assays using vivid fluorescent substrates and recombinant human cytochrome P450 enzymes.. J Biomol Screen.

[21] Marks BD, Thompson DV, Goossens TA, Trubetskoy OV (2004). High-throughput screening assays for the assessment of CYP2C9*1, CYP2C9*2, and CYP2C9*3 metabolism using fluorogenic Vivid substrates.. J Biomol Screen.

[22] Marks BD, Smith RW, Braun HA, Goossens TA, Christenson M (2002). A high throughput screening assay to screen for CYP2E1 metabolism and inhibition using a fluorogenic vivid p450 substrate.. Assay Drug Dev Technol.

[23] Marks BD, Goossens TA, Braun HA, Ozers MS, Smith RW (2003). High-throughput screening assays for CYP2B6 metabolism and inhibition using fluorogenic vivid substrates.. AAPS PharmSci.

[24] Cohen LH, Remley MJ, Raunig D, Vaz AD (2003). In vitro drug interactions of cytochrome p450: an evaluation of fluorogenic to conventional substrates.. Drug Metab Dispos.

[25] Trubetskoy O, Marks B, Zielinski T, Yueh MF, Raucy J (2005). A simultaneous assessment of CYP3A4 metabolism and induction in the DPX-2 cell line.. Aaps J.

[26] Patel J, Buddha B, Dey S, Pal D, Mitra AK (2004). In vitro interaction of the HIV protease inhibitor ritonavir with herbal constituents: changes in P-gp and CYP3A4 activity.. Am J Ther.

[27] Ansede JH, Thakker DR (2004). High-throughput screening for stability and inhibitory activity of compounds toward cytochrome P450-mediated metabolism.. J Pharm Sci.

[28] He N, Edeki T (2004). The inhibitory effects of herbal components on CYP2C9 and CYP3A4 catalytic activities in human liver microsomes.. Am J Ther.

[29] Ji HY, Lee HW, Kim HK, Kim HH, Chang SG (2004). Simultaneous determination of ginsenoside Rb(1) and Rg(1) in human plasma by liquid chromatography-mass spectrometry.. J Pharm Biomed Anal.

[30] Wang H, Zou H, Kong L, Zhang Y, Pang H (1999). Determination of ginsenoside Rg3 in plasma by solid-phase extraction and high-performance liquid chromatography for pharmacokinetic study.. J Chromatogr B Biomed Sci Appl.

[31] Wang RW, Newton DJ, Liu N, Atkins WM, Lu AY (2000). Human cytochrome P-450 3A4: in vitro drug-drug interaction patterns are substrate-dependent.. Drug Metab Dispos.

[32] Nasrallah HA, Ketter TA, Kalali AH (2006). Carbamazepine and valproate for the treatment of bipolar disorder: a review of the literature.. J Affect Disord.

[33] Peebles CT, McAuley JW, Roach J, Moore JL, Reeves AL (2000). Alternative Medicine Use by Patients with Epilepsy.. Epilepsy Behav.

[34] Stresser DM, Blanchard AP, Turner SD, Erve JC, Dandeneau AA (2000). Substrate-dependent modulation of CYP3A4 catalytic activity: analysis of 27 test compounds with four fluorometric substrates.. Drug Metab Dispos.

[35] Racha JK, Zhao ZS, Olejnik N, Warner N, Chan R (2003). Substrate dependent inhibition profiles of fourteen drugs on CYP3A4 activity measured by a high throughput LCMS/MS method with four probe drugs, midazolam, testosterone, nifedipine and terfenadine.. Drug Metab Pharmacokinet.

[36] Anger DL, Petre MA, Crankshaw DJ (2005). Heteroactivation of cytochrome P450 1A1 by teas and tea polyphenols.. Br J Pharmacol.

[37] Nakamura H, Nakasa H, Ishii I, Ariyoshi N, Igarashi T (2002). Effects of endogenous steroids on CYP3A4-mediated drug metabolism by human liver microsomes.. Drug Metab Dispos.

[38] Nakamura H, Torimoto N, Ishii I, Ariyoshi N, Nakasa H (2003). CYP3A4 and CYP3A7-mediated carbamazepine 10,11-epoxidation are activated by differential endogenous steroids.. Drug Metab Dispos.

[39] Yano JK, Wester MR, Schoch GA, Griffin KJ, Stout CD (2004). The structure of human microsomal cytochrome P450 3A4 determined by X-ray crystallography to 2.05-A resolution.. J Biol Chem.

[40] Hosea NA, Miller GP, Guengerich FP (2000). Elucidation of distinct ligand binding sites for cytochrome P450 3A4.. Biochemistry.

[41] Roberts AG, Atkins WM (2007). Energetics of heterotropic cooperativity between alpha-naphthoflavone and testosterone binding to CYP3A4.. Arch Biochem Biophys.

[42] Cameron MD, Wen B, Allen KE, Roberts AG, Schuman JT (2005). Cooperative binding of midazolam with testosterone and alpha-naphthoflavone within the CYP3A4 active site: a NMR T1 paramagnetic relaxation study.. Biochemistry.

[43] Lu P, Lin Y, Rodrigues AD, Rushmore TH, Baillie TA (2001). Testosterone, 7-benzyloxyquinoline, and 7-benzyloxy-4-trifluoromethyl-coumarin bind to different domains within the active site of cytochrome P450 3A4.. Drug Metab Dispos.

[44] Torimoto N, Ishii I, Hata M, Nakamura H, Imada H (2003). Direct interaction between substrates and endogenous steroids in the active site may change the activity of cytochrome P450 3A4.. Biochemistry.

[45] Jiang X, Williams KM, Liauw WS, Ammit AJ, Roufogalis BD (2004). Effect of St John's wort and ginseng on the pharmacokinetics and pharmacodynamics of warfarin in healthy subjects.. Br J Clin Pharmacol.

[46] Gurley BJ, Gardner SF, Hubbard MA, Williams DK, Gentry WB (2002). Cytochrome P450 phenotypic ratios for predicting herb-drug interactions in humans.. Clin Pharmacol Ther.

